# [Ti(py)_4_I_2_] as an Entry Point to Titanium(II) Chemistry via Titanium Metal Oxidation

**DOI:** 10.1002/chem.70955

**Published:** 2026-04-03

**Authors:** Alexander Sedykh

**Affiliations:** ^1^ Institute of Inorganic Chemistry and Crystallography Leipzig University Leipzig Germany; ^2^ Institute of Inorganic and Analytical Chemistry Justus‐Liebig‐University Giessen Giessen Germany

**Keywords:** halides, n ligands, synthesis design, titanium

## Abstract

Access to defined titanium(II) compounds is crucial for their use in synthesis and catalysis. Most established routes to Ti(II) compounds rely on the reduction of Ti(III) or Ti(IV), an approach that intrinsically limits atom efficiency and can introduce byproducts or lead to mixed‐valency complexes. Here, an alternative strategy for obtaining Ti(II) is presented, based on the oxidation of Ti metal by iodine. Ti diiodide formed in this reaction is subsequently complexed to afford the novel Ti(II) complex [Ti(py)_4_I_2_] on a gram scale (> 30 g, 50 mmol) in a highly atom‐efficient approach. Revision of the original synthesis of TiI_2_ shows that excess Ti metal is required to prevent the formation of higher Ti iodides, enabling clean access to Ti(II) without reductive agents. The resulting Ti(II) complex serves as a synthetic entry point for Ti(II) chemistry, as illustrated by a ligand exchange reaction to form [Ti(2,2′‐bipyridine)_2_I_2_].

## Graphical abstract

A strategically novel approach based on the oxidation of Ti metal enables access to Ti(II) chemistry. Controlled formation and complexation of Ti diiodide affords the Ti(II) complex [Ti(py)_4_I_2_], which serves as a convenient entry point to Ti(II) compounds.

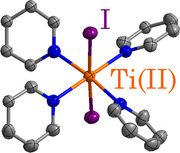

## 1 Introduction

Ti(II) compounds play a significant role in catalysis, especially in the activation of double and triple bonds in organic molecules [1, 2]. Ti(II) complexes are also effective for small‐molecule activation [3, 4, 5, 6, 7, 8]. Due to its high elemental abundance [1] and being a noncritical element [9], Ti(II) can also be used stoichiometrically [10]. Yet, the use of Ti(II) is limited by the lack of practical synthetic entry points. The drawback of the typical approach based on the reduction of Ti(III)/Ti(IV) to form catalytically active Ti(II) species [2, 9] is the formation of reduction by‐products [11, 12, 13, 14], which decreases the purity of the catalytic system and inherently limits atom efficiency. To date, no general alternative strategy based on the oxidation of Ti metal to access Ti(II) coordination compounds has been established.

Arguably, Ti(II) dihalides represent the most suitable starting materials for the synthesis of Ti(II) coordination compounds. They are obtained by reacting Ti with TiF_3_, TiCl_4_, Br_2_, or I_2_ [15, 16, 17, 18]. For TiCl_2_ and TiBr_2_, direct complexation with acetonitrile afforded coordination compounds that were subsequently used as synthetic precursors of Ti(II) compounds [19]. However, their crystal structures were not investigated at the time and remain unreported. In later studies, the coordination compounds of Ti(II) were obtained almost exclusively by reduction of Ti(III) or Ti(IV) complexes.

To obtain Ti(II), higher oxidation states of Ti are reduced by magnesium [20, 21, 22, 23, 24, 25, 26, 27, 28, 29, 30, 31, 32, 33, 34, 35], alkali metals or their alloys [8, 12, 36, 37, 38, 39, 40], KC_8_ [41, 42, 43, 44], carbon monoxide [45], hydrogen [3], organolithium compounds [46, 47, 48, 49], Grignard reagents [50, 51, 52], complex hydrides [10, 13, 53, 54], or aluminium [55, 56]. Despite their applicability, these reductive approaches have some intrinsic limitations. The requirement for stoichiometric reducing agents inherently compromises atom efficiency and often necessitates extensive workup and purification. In addition, incomplete reduction can lead to the formation of Ti(III) or mixed‐valent Ti(III)/Ti(II) species [8, 14, 30], whereas overly strong reducing conditions may result in overreduction to Ti(0) [57]. Finally, incorporation of residual reducing agents or their by‐products into the final Ti(II) compounds cannot always be excluded [11, 12, 13, 14], as exemplified by the formation of intermetallic species such as [CpTi(μ‐btmse)_2_MgCp] (btmse = bis(trimethylsilyl)ethyne) [11].

Besides the early examples of acetonitrile adducts TiCl_2_·2MeCN and TiBr_2_·2MeCN and complexes derived from them (with no crystal structure reported) [19], only a small number of Ti(II) complexes have been obtained via oxidation of Ti(0). However, these synthetic methods are impractical due to the very low single‐digit yield [58] or susceptibility to overoxidation of Ti under the reaction conditions [59].

In this work, a practical oxidative route to Ti(II) chemistry is introduced. Oxidation of Ti metal with iodine, followed by direct complexation with pyridine, yields [Ti^II^(py)_4_I_2_]·2Py (**1**). Clean formation of Ti diiodide requires an excess of Ti, which can be reused after separation. Subsequent removal of intercalated solvent affords the final complex [Ti^II^(py)_4_I_2_] (**2**), which is obtained quantitatively with high atom efficiency on a 50 mmol scale (30.5 g) from starting materials that are handled in air. As no external reducing agents are employed, contamination by reductant‐derived by‐products is excluded. The resulting Ti(II) complex serves as a convenient synthetic entry point to Ti(II) chemistry, as demonstrated by ligand‐exchange formation of [Ti^II^(bpy)_2_I_2_] (**3**) (bpy = 2,2′‐bipyridine).

## 2 Results and Discussion

### 2.1 Synthesis of a Novel Titanium(II) Source and Its Exemplary Usage

**SCHEME 1:**
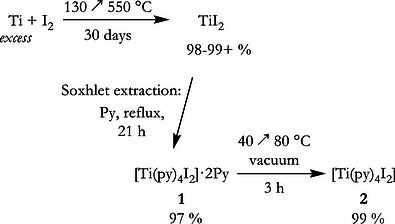
Synthesis of TiI_2_ (in a mixture with an excess of Ti metal), [Ti^II^(py)_4_I_2_]·2Py (**1**), and [Ti^II^(py)_4_I_2_] (**2**). Overall yields are shown.

Oxidative access to Ti(II) was achieved through a reaction of Ti metal with iodine, in which the Ti diiodide was obtained as a key Ti(II) precursor (Scheme 1). It was found that an excess of Ti metal is essential to obtain Ti diiodide free of impurities of higher Ti iodides. When the equimolar ratio of Ti and iodine is used, a mixture of TiI_2_ and TiI_3_ was unexpectedly obtained (Figure S1). When the Ti excess is used, phase‐pure Ti diiodide is obtained (Figure S2). The excess metal can be separated (Figure S3) and reused, establishing this oxidative step as a clean and controllable entry to Ti(II).

The complexation of TiI_2_ and its separation was achieved simultaneously by pyridine in the Soxhlet extraction, resulting in the formation of [Ti(py)_4_I_2_]·2Py (**1**) (Scheme 1). This hybrid approach affords the Ti(II) complex **1** in pure form (Figure S4), quantitatively, avoiding additional purification steps. The removal of the intercalated solvent yields the stable complex [Ti(py)_4_I_2_] (**2**) (Scheme 1). The starting materials are easily handled in air, whereas the Ti(II) products [Ti(py)_4_I_2_]·2Py (**1**) and [Ti(py)_4_I_2_] (**2**) are oxygen‐ and moisture‐sensitive. Therefore, during the synthesis, the colour change could easily indicate whether these complexes were oxidised. In the powder form, both complexes have a deep blue colour, appearing even black. Their solution in pyridine also has a deep blue colour, and if it is slightly exposed to oxygen or moisture, it takes on a green tint. The same solution colour change was also previously observed for TiCl_2_·2Py [19], however, at that time, it was not associated with the oxidation of Ti(II). Fully, when the [Ti(py)_4_I_2_] (**2**) is exposed to air, the solution rapidly turns yellow (see photos in the Supporting Information).

Complex **2** was obtained on a 50 mmol scale (30.5 g) in a quantitative yield and phase‐pure form (Figure S5) with high atom efficiency. In comparison to other isolable Ti(II) sources—[Ti^II^(tmeda)_2_Cl_2_] (70 mmol, 85%) [36], [Ti^II^(dmpe)_2_Cl_2_] (6.4 mmol, 70%) [20], and [Ti^II^Cp_2_(PMe_3_)_3_] (40 mmol, 99+%) [21]—the present approach avoids using stoichiometric reducing agents and correlated workup, while also relying on inexpensive and nonhazardous ligand.

**SCHEME 2:**
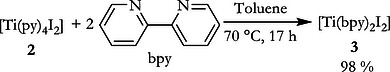
Synthesis of [Ti(bpy)_2_I_2_] (3).

The suitability of **2** as a synthetic Ti(II) source was demonstrated in a ligand exchange reaction with 2,2′‐bipyridine (bpy), which proceeds to afford [Ti(bpy)_2_I_2_] (**3**) (Scheme 2). The quantitative conversion to the phase pure **3** (Figure S6), driven by the differences in solubility (Table 1), confirms the lability of pyridine ligands in **2** and that this complex serves as an entry point to Ti(II) coordination chemistry.

**TABLE 1:** Comparison of the solubilities and stability of **2** and **3** in common organic solvents.

Solvents	[Ti(py)_4_I_2_] (2)	[Ti(bipy)_2_I_2_] (3)
Pyridine	Well soluble^a^	Well soluble^b^
Toluene	Soluble	Insoluble
Tetrahydrofuran	Well soluble	Insoluble
Diethyl ether	Insoluble	Insoluble
Acetonitrile	Reacts^c^	Soluble
Dimethylformamide	Reacts^c^	Soluble
Acetone	Reacts^c^	Reacts^d^
Chloroform	Reacts^c^	Insoluble
Hexane	Insoluble	Insoluble
Ethyl acetate	Insoluble	Insoluble

^a^ Maximum concertation 1.7(2) mM (≈1 mg · mL^−1^) as estimated by UV–vis‐NIR spectroscopy.

^b^ Solubility is at least 3 mM (>1.8 mg · mL^−1^).

^c^ Evidenced by the formation of a red solution that does not change color on contact with air.

^d^ Evidenced by the formation of a green solution that does not change color on contact with air.

### 2.2 Structural Analysis

**FIGURE 1:**
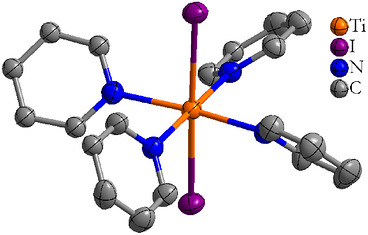
Selected view of X‐ray crystal structure of [Ti(py)_4_I_2_] (**2**). Thermal ellipsoids describe a 50% probability level of the atoms (Ti orange, I violet, C grey, and N blue); hydrogen atoms are omitted.

Compound [Ti(py)_4_I_2_]·2Py (**1**) crystallises in *Ccca*, while after the intercalated solvent removal [Ti(py)_4_I_2_] (**2**) has a space group *Pbcn*. In both compounds, the Ti centre adopts an elongated octahedral coordination geometry defined by two iodide ligands in a *trans* position and four equatorial pyridine donors (Figure 1). The molecular unit in **1** and **2** has an inversion point, making them, in principle, nonpolar. Complex **1** is isostructural to related [TM^II^(py)_4_I_2_]·2Py [60, 61] and complex **2** to [TM^II^(py)_4_I_2_] [62, 63]. In **1** and **2**, the interatomic distances Ti‐I (2.8960(3)–2.9259(2) Å) are significantly longer than those reported for Ti^III^‐I (2.7508–2.7699 Å) [59, 64] and Ti^IV^‐I (2.6904–2.8013 Å) [65, 66], and match Ti^II^‐I in a sole reported coordination compound (3.0541 Å) [59] and in TiI_2_ (2.8899(4) Å). For comparison, in other inorganic Ti iodides, interatomic distances Ti‐I are shorter, namely 2.7783(2) Å in TiI_3_ and 2.5327(5)/2.5363(3) Å in TiI_4_, determined from single crystal diffraction data measured for this work for better comparison.

Unlike ionic Ti‐I distances, covalent Ti‐N_Py_ are less dependent on the oxidation state of Ti: Ti^II^‐N_py_ 2.165–2.280 Å [40, 41, 44], Ti^III^‐N_py_ 2.249–2.387 Å [44], and Ti^IV^‐N_py_ 2.225–2.450 [44, 67], with an overlap in ranges and only a slight increase in ranges as going from low to high oxidation state of Ti. In both **1** and **2**, the interatomic distances Ti‐N_py_ are 2.243(4)–2.278(2) Å, corresponding to the typical values for Ti‐N_Py_. To conclude the comparison, for complexes **1** and **2**, interatomic distances between the metal and ligand atoms correspond to the expected values for Ti(II) complexes.

**FIGURE 2:**
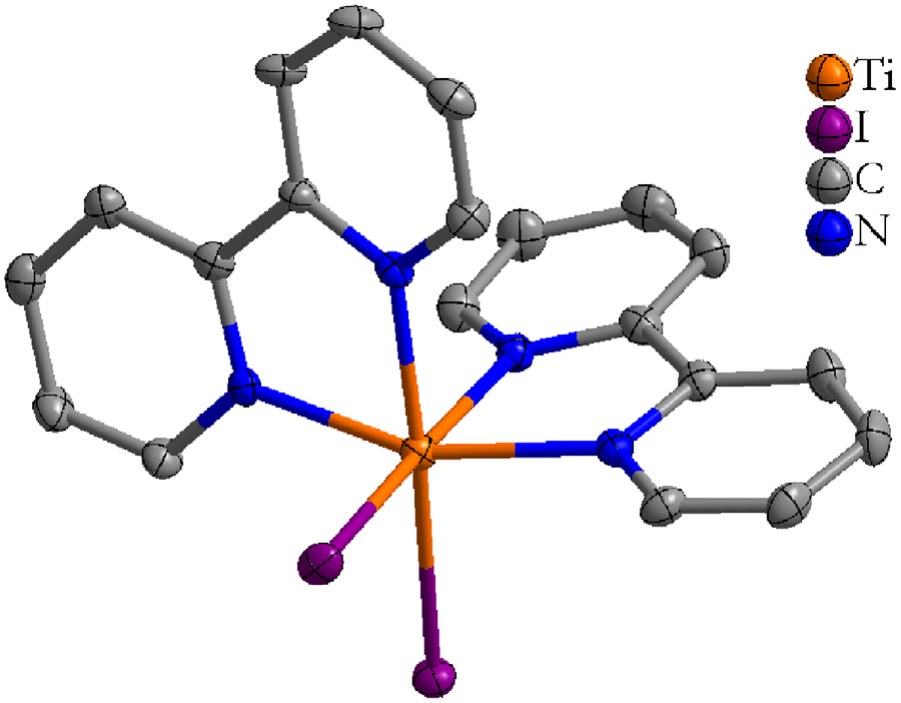
Selected view of X‐ray crystal structure of [Ti(bpy)_2_I_2_] (**3**). Thermal ellipsoids describe a 50% probability level of the atoms (Ti orange, I violet, C grey, and N blue); hydrogen atoms are omitted.

Complex [Ti(bpy)_2_I_2_] (**3**) crystallises in *P*2_1_/*c*, with coordinated to the metal iodides being in *cis* position, and two bpy ligands coordinating with two nitrogen atoms each (Figure 2). Compound **3** is isostructural to [Mn(bpy)_2_Br_2_] [68]. For **3**, the interatomic distances Ti‐I are 2.7572(9)‐2.7705(2) Å and Ti‐N_bpy_ are 2.109(4)‐2.142(2) Å. The interatomic distances Ti‐I in **3** are shorter than in **1** and **2** (2.8960(3)‐2.9259(2) Å) and the only other example in the literature for Ti^II^‐I (3.0541 Å) [59]. Therefore, for the comparative crystallographic analysis of **3**, several complexes with Ti(III) iodide and bpy were obtained: [Ti^III^(bpy)_2_I_2_]I (**4**), [Ti^III^(bpy)_2_I_2_]I·0.5bpy (**5**), and [Ti^III^(bpy)_2_ClI]I·CHCl_3_ (**6**). In **4** and **5**, the molecular unit is practically the same as in **3**, with the third iodine being non‐coordinating. In **6**, both halides coordinated to the Ti atom are disordered, and on both crystallographic positions a mixture of Cl/I is present with a ratio of 0.27/0.73 and an inverted 0.73/0.23. For **4** and **5**, interatomic distances Ti‐I are 2.694(2)‐2.706(2) Å. For **6**, the Ti‐I distances are 2.649(3) and 2.7719(8) Å, with a difference from those of **4** and **5** likely due to the disorder handling of the data. Although the average Ti‐I interatomic distance is 2.71 Å in **6**, corresponding well with the values for **4** and **5**. Altogether, for the three Ti(III) compounds **4**–**6**, the interatomic distance Ti‐I is noticeably shorter than in **3**, suggesting that the latter has a lower oxidation state of Ti.

Another evidence that the complex **3** has Ti(II) is the intact structure of the organic ligand. In one previous report on the interaction of Ti(II) with bpy it was disputed that this organic ligand can be reduced by Ti(II) with the formation of the anionic radical bpy^•–^ being coordinated to the forming Ti(III) [69]. This is indicated by the change in the structure of the ligand itself, where the interatomic distance 2,2′‐C‐C (between the pyridine rings) shortens from 1.480 Å (for the free bpy) to 1.416 Å, and 1,2‐N‐C and 1′,2′‐N‐C are increased from 1.36 to 1.40 Å [69]. For **3**, interatomic distances 2,2′‐C‐C are 1.454(7) and 1.463(7) Å, and 1,2‐/1′,2′‐N‐C are 1.363(6)–1.373(6) Å. For **4**–**6**, interatomic distances 2,2′‐C‐C are 1.45(2)–1.48(2) Å, 1,2‐/1′,2′–N‐C are 1.349(8)–1.368(4) Å. Evidently, for all compounds **3**–**6**, there is no change in the structure of the ligand present, which leads to the conclusion that bpy is neutral. Therefore, an electron transfer from the Ti metal ion to the ligand can be excluded. Based on this, the oxidation state of Ti in complexes **3**–**6** can be assumed as nominal, being Ti(II) for [Ti(bpy)_2_I_2_] (**3**) and Ti(III) for **4**–**6**. In the octahedral crystal field, Ti(II) with a d^2^ configuration will have a *t_2g_^2^* configuration in complexes **1**–**3**, and Ti(III), d^1^, will have a *t_2g_^1^* configuration in complexes **4**–**6**, all six complexes being paramagnetic.

Details on the crystallographic data for compounds **1**–**6**, as well as TiI_2_, Ti_3_, and TiI_4_, are presented in the Supporting Information (Tables S1–S3). Interatomic distances and angles for Ti atoms coordination spheres for compounds **1**–**6**, as well as TiI_2_, Ti_3_, and TiI_4_, are also presented in the Supporting Information (Tables S4–S6), together with views of X‐ray crystal structures (Figures S7–S9).

### 2.3 Optical Spectroscopy and Thermal Analysis

**FIGURE 3:**
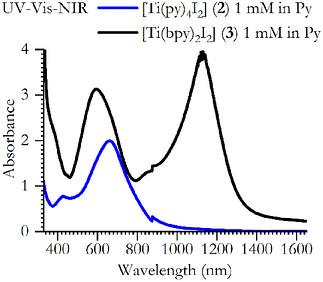
UV–vis‐NIR absorbance spectra of **2** (blue line) and **3** (black line), recorded for 1 mm solutions in dry pyridine in 10 mm cuvettes.

Compounds **2** and **3** have intense, broad absorption peaks, measured in pyridine (Figure 3), with molar extinction coefficients reaching ≈2000 M^−1^ · cm^−1^ at 660 nm for [Ti(py)_4_I_2_] (**2**) and ≈3000 M^−1^ · cm^−1^at 595 and 1125 nm for [Ti(bpy)_2_I_2_] (**3**) (Figures S10–S14). The absorption spectra correspond to the colours of solutions: dark blue for **2** and black for **3**. Based on optical absorption spectroscopy, the maximum solubility of **2** in pyridine was estimated to be ≈1 mg · mL^−1^ (Figure S12).

**FIGURE 4:**
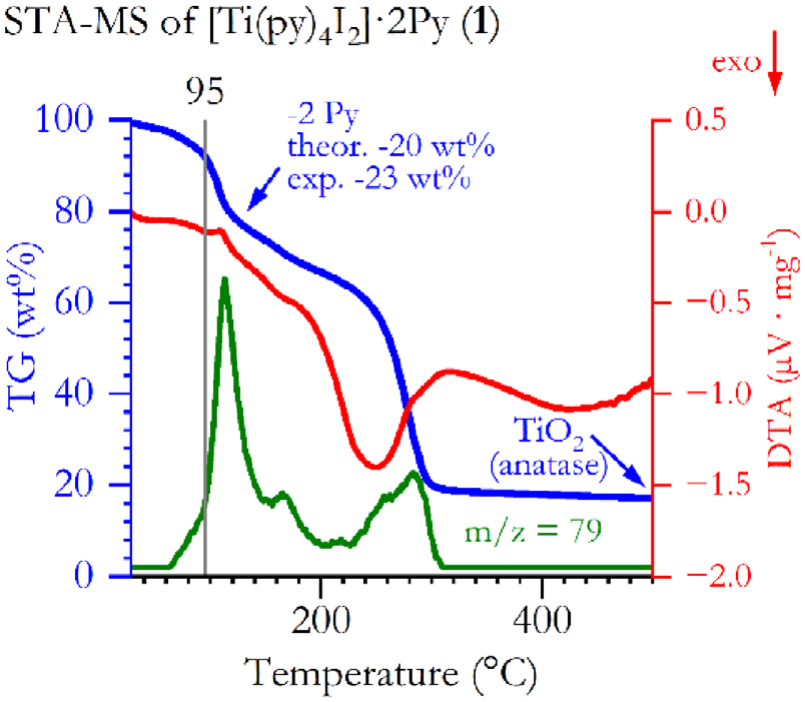
Simultaneous thermal analysis and mass spectrometry of **1**, measured under Ar. Thermogravimetry (TG, blue), differential thermal analysis (DTA, heat flow, red), and ionic current of mass‐spectrometer for *m/z* = 79 (Py^+^, green) are shown.

The intercalated pyridine is released from [Ti(py)_4_I_2_]·2Py (**1**) with *T*_onset_ 95°C, the process being endothermic (Figure 4). The resulting [Ti(py)_4_I_2_] (**2**) undergoes decomposition in an overlapping process. Despite the measurement being performed under inert conditions, the final compound identified by PXRD is anatase. This observation highlights the extreme sensitivity of Ti(II) species to oxidation, with oxygen likely originating from trace impurities in the argon atmosphere or from a reaction with the corundum crucible. During the synthesis of **2**, the release of intercalated pyridine from **1** was achieved at a lower temperature of 80°C, avoiding decomposition or oxidation of the desired product.

## 3 Conclusion

A practical, atom‐efficient oxidative route to Ti(II) chemistry has been established using Ti metal as the starting material. The Ti(II) complex [Ti(py)_4_I_2_] (**2**) was obtained on a gram scale (50 mmol, 30.5 g) in quantitative yield without the use of reductive agents, avoiding limitations associated with reduction‐based access to Ti(II), such as byproduct formation or mixed‐valent species. The complex **2** serves as a convenient and well‐defined synthetic entry point to Ti(II) coordination chemistry, as demonstrated by clean ligand‐exchange formation of [Ti(bpy)_2_I_2_] (**3**). Structural analysis confirms the Ti(II) oxidation state for the compounds presented. This work establishes oxidative access to Ti(II) as a viable and scalable alternative to reductive strategies, opening new opportunities for the preparation and application of Ti(II) compounds.

## Conflicts of Interest

The authors declare no conflicts of interest.

## Supporting information




**Supporting File**: Additional references have been cited within the Supporting Information [70, 71, 72, 73]. Supporting Information (16 pages) to this manuscript contains experimental details and data, figures with comparison of measured and simulated powder X‐ray diffraction patterns, tables with X‐ray crystallographic data, tables with selected interatomic distances and angles, figures with UV–vis‐NIR absorbance spectra, and photos of selected experimental setups used in syntheses and photos showing the sensitivity of [Ti^II^(py)_4_I_2_] (**2**) toward air. Deposition numbers 2513455 (for [Ti(py)_4_I_2_]·2Py (**1**)), 2513456 (for [Ti(py)_4_I_2_] (**2**)), 2513457 (for [Ti(bpy)_2_I_2_] (**3**)), 2513458 (for [Ti(bpy)_2_I_2_]I (**4**)), 2513459 (for [Ti(bpy)_2_I_2_]I·0.5bpy (**5**)), 2513460 (for [Ti(bpy)_2_ClI]I·CHCl_3_ (for **6**)), 2513461 (for TiI_2_), 2513462 (for TiI_3_), and 2513463 (for TiI_4_) contain the supplementary crystallographic data for this paper. These data are provided free of charge by the joint Cambridge Crystallographic Data Centre and Fachinformationszentrum Karlsruhe Access Structures service.

## Data Availability

The data that support the findings of this study are available from the corresponding author upon reasonable request.
