# Supplementary material for: [Ti(py)4I2] as an Entry Point to Titanium(II) Chemistry via Titanium Metal Oxidation
Source: Chemistry. 2026 Apr 3;32(23):e70955. doi: 10.1002/chem.70955 (PMC13282914; doi:10.1002/chem.70955)
Supplement: Supplementary file 1 — Supporting File: Additional references have been cited within the Supporting Information [70, 71, 72, 73]. Supporting Information (16 pages) to this manuscript contains experimental details and data, figures with comparison of measured and simulated powder X‐ray diffraction patterns, tables with X‐ray crystallographic data, tables with selected interatomic distances and angles, figures with UV–vis‐NIR absorbance spectra, and photos of selected experimental setups used in syntheses and photos showing the sensitivity of [TiII(py)4I2] (2) toward air. Deposition numbers 2513455 (for [Ti(py)4I2]·2Py (1)), 2513456 (for [Ti(py)4I2] (2)), 2513457 (for [Ti(bpy)2I2] (3)), 2513458 (for [Ti(bpy)2I2]I (4)), 2513459 (for [Ti(bpy)2I2]I·0.5bpy (5)), 2513460 (for [Ti(bpy)2ClI]I·CHCl3 (for 6)), 2513461 (for TiI2), 2513462 (for TiI3), and 2513463 (for TiI4) contain the supplementary crystallographic data for this paper. These data are provided free of charge by the joint Cambridge Crystallographic Data Centre and Fachinformationszentrum Karlsruhe Access Structures service. [file CHEM-32-e70955-s001.pdf]

Supporting Information for

[Ti(py)$_4$I$_2$] as an Entry Point to Titanium(II) Chemistry via Titanium Metal Oxidation

A. Sedykh

Table of Contents

S1. Experimental details and data 2
S1.1. Methods and materials 2
S1.2. Analytical Methods 2
S1.3. Bulk material synthesis 2
S1.4. Single crystal synthesis 4
S2. Crystallographic data 5
S2.1. Comparison of measured and simulated powder X-ray diffraction patterns 5
Figure S1. Experimental powder X-ray diffraction pattern of the reaction mixture from the synthesis of TiI$_2$ 5
Figure S2. Experimental powder X-ray diffraction pattern of the reaction mixture from the synthesis of TiI$_2$ 5
Figure S3. Experimental powder X-ray diffraction pattern of residuals in the thimble after Soxhlet extraction 5
Figure S4. Comparison of experimental (top) and simulated (bottom) powder X-ray diffraction patterns of [Ti(py)$_4$I$_2$]·2Py (**1**) 6
Figure S5. Comparison of experimental (top) and simulated (bottom) powder X-ray diffraction patterns of [Ti(py)$_4$I$_2$] (**2**) 6
Figure S6. Comparison of experimental (top) and simulated (bottom) powder X-ray diffraction patterns of [Ti(bpy)$_2$I$_2$] (**3**) 6
S2.2. Tables with X-ray crystallographic data 7
Table S1. Crystallographic data for [Ti(py)$_4$I$_2$]·2Py (**1**), [Ti(py)$_4$I$_2$] (**2**), and [Ti(bpy)$_2$I$_2$] (**3**) 7
Table S2. Crystallographic data for [Ti(bpy)$_2$I$_2$]I (**4**), [Ti(bpy)$_2$I$_2$]I·bpy (**5**), and [Ti(bpy)$_2$ClI]I·CHCl$_3$ (**6**) 8
Table S3. Crystallographic data for TiI$_2$, TiI$_3$, TiI$_4$ 9
S2.3. Tables with selected interatomic distances and angles 10
Table S4. Selected interatomic distances (Å) and angles (°) of [Ti(py)$_4$I$_2$]·2Py (**1**) and [Ti(py)$_4$I$_2$] (**2**) 10
Table S5. Selected interatomic distances (Å) and angles (°) of [Ti(bpy)$_2$I$_2$] (**3**), [Ti(bpy)$_2$I$_2$]I (**4**), [Ti(bpy)$_2$I$_2$]I·bpy (**5**), and [Ti(bpy)$_2$ClI]I·CHCl$_3$ (**6**) 11
Table S6. Selected interatomic distances (Å) and angles (°) of TiI$_2$, TiI$_3$, TiI$_4$ 12
S3. UV-Vis-NIR absorbance spectra 13
S4. Photos of selected experimental setup and reaction of [Ti(py)$_4$I$_2$] (2) solution with air 15
S4.1. Photo of the experimental setup used for the complexation/Soxhlet extraction of [Ti(py)$_4$I$_2$] 15
S4.2. Reaction of [Ti(py)$_4$I$_2$] (**2**) solution with air 16
S5. References 16

S1. Experimental details and data

S1.1. Methods and materials

General information. Quartz tubes (outer diameter 18 mm, wall thickness 2 mm, length till a constriction ≈17 cm, total length ≈25 cm) that were used to make ampoules for the synthesis of titanium iodides were prepared by professional glassblowers. A glovebox (MBraun Labmaster SP), vacuum gas manifold with valves with PTFE-spindles, self-made quartz tubes for the ampoule preparation (outer diameter 10 mm, wall thickness 1 mm, length after sealing ≈10 cm), self-made Duran glass tubes for the ampoule preparation (outer diameter 10 mm, wall thickness 1.5 mm, length after sealing ≈10 cm), Duran culture tubes, and special quick-fits for connecting glass tubes to the vacuum gas manifold were used in the syntheses. A special cutoff adapter was used, consisting of the conical joint NS29/32 cone, a straight high-vacuum valve with a PTFE spindle (passage 0-15 mm), and a conical joint NS29/32 socket (from bottom to the top, see SI Figure S16 for a photo, part c). Products were stored and prepared for the analytics inside the glovebox.

Starting materials. Titanium powder (99.9 %, -150 mesh, Alfa Aesar), titanium sponge (99.95 %, 3-13 mm, Alfa Aesar), iodine (≥99 %), 2,2-bipyridine (≥99 %, Sigma-Aldrich), ZnI_2 (98 %, Acros), were used as received. The benzene was dried using standard techniques and stored over molecular sieves inside the glass with a valve with PTFE-spindle. Other dry solvents (Thermo Scientific, AcroSeal™, with molecular sieves unless otherwise stated), namely pyridine (99.5 %), toluene (99.85 %), acetonitrile (99.9 %, without molecular sieves), chloroform (99.9 %), and diethyl ether (99.5 %) were used as received.

S1.2. Analytical Methods

Single crystal X-ray diffraction analysis (SCXRD). Inside the glovebox, single crystals were placed on the object glass and submerged in a perfluorinated ether. The crystals were transported to the diffractometer in a Schlenk vessel and selected for the measurement in a counterstream of nitrogen gas. Data collections were performed using Mo-Kα (for TiI_2, TiI_3, TiI_4, **1**, **3**, **4**, **5**, **6**) or Cu-Kα (for **2**) X-ray radiation. Measurements were done with a BRUKER AXS D8 Venture diffractometer with an IμS microfocus source and a PHOTON100 detector at 100 K (for TiI_2, TiI_3, TiI_4, **1**, **3**), 120 K (**4** and **6**), 150 K (for **5**), or 200 K (for **2**). For the data collection, the BRUKER AXS Apex software was used. Data processing was accomplished with XPREP, structure solutions were carried out with direct methods using SHELXT,[70] and the obtained crystal structures were refined with least square techniques using SHELXL[71] on the graphical platform shelXle.[72]

Powder X-ray diffraction analysis (PXRD). Inside a glovebox, a sample was filled in a glass mark tube (Ø 0.3 mm, Hilgenberg GmbH), which was cut and sealed with a picein wax. Diffraction data were collected in a transmission geometry with a powder X-ray diffractometer STOE Stadi P equipped with a focusing Ge(111) monochromator and a MYTHEN 1K strip detector (angular range 12.5° in 2θ) using Cu-Kα X-ray radiation. The collection was done in a 2θ range 5-60° for **1**-**3** or 5-90° for TiI_2, with a step size of 0.015° and an integration time of 20 s.

Elemental analysis. For CHN analysis, the compounds were placed in a tin crucible inside a glovebox. Analyses were done with a Vario Micro Cube.

Ultraviolet-visible-near infrared spectroscopy (UV-Vis-NIR). Solutions for absorption spectra measurement were prepared inside the glovebox. For **2**, solutions of 1, 0.8, and 0.5 mM were prepared directly by dissolving the corresponding amount of the compound in dry pyridine. A concentrated solution of **2** was filtered through a PTFE syringe filter before measurement. For **3**, firstly, the 3 mM solution was prepared directly by dissolving a corresponding amount of the compound in dry pyridine, and solutions of 2, 1, and 0.5 mM were prepared by mixing the 3 mM solution with dry pyridine. Absorption spectra have been recorded using a Cary 5000 Series UV-Vis-NIR spectrophotometer (Agilent Technologies) in double beam mode using Cary WinUV software (Agilent Technologies). Measurements were acquired at room temperature for solutions filled in cuvettes with an optical pathway of 10 mm, recoded from 1650 to 330 nm with a step of 1 nm, a scan rate of 600 nm · min^{-1}, a spectral bandwidth of 2 nm, and with the detector and monochromator grating changeover at 875 nm.

Thermal analysis (STA-MS). Simultaneous differential thermal analysis and thermogravimetry (DTA/TG or STA, thermocouple type S) were performed using a NETZSCH STA 409-PC thermal analyser coupled with a QMS 403 Aëolos mass spectrometer (MS). Measurement for **1** was performed for 14.1 mg of the sample in a corundum crucible (prepared in a glovebox) with a lid in a flow of argon (30 mL · min^{-1} working gas, 20 mL · min^{-1} protective gas) with a heating rate of 5 K · min $^{-1}$ from room temperature to 500 °C. Residuals from the measurement were analysed by PXRD analysis.

S1.3. Bulk material synthesis

Synthesis of TiI_2 (in a mixture with an excess of Ti metal). The synthesis is a modification of a literature procedure.[15,16] Prior to the reaction, the quartz tube (prepared by a professional glassblower) was stored in a drying cabinet (110 °C). The quartz tube was fixed in a quick-fit and flushed with argon three times using the vacuum gas manifold. Then, iodine (12.69 g, 50.0 mmol) was added to the tube once it cooled down to room temperature. The titanium powder (3.595, 75.0 mmol) was weighed in the glovebox and then added to the tube using a long funnel. After that, the tube was promptly evacuated and flushed with argon. Before sealing the ampoule, the tube was again promptly evacuated, and the bottom part with chemicals was cooled with liquid nitrogen. After that, the tube was evacuated to 0.5 Pa and sealed while still being cooled with liquid nitrogen. Upon cooling, the ampoule showed dark orange rings above the iodine level, which vanished once the ampoule was warmed to room temperature. The resulting ampoule (≈17 cm) was placed vertically in the middle of a tube furnace (total length 36 cm) controlled by a Eurotherm 2416 PID controller. The bottom and top of the oven tube were filled with stone wool, and both exits were covered by anti-explosion protection. First, the mixture was pre-reacted according to the following heating program (sum time 6 days): (1a) heating to 130 °C in 2 h; (1b) dwelling for 22 h; (1c) heating to 180 °C in 2 h; (1d) dwelling for 46 h; (1e) heating to 400 °C in 6 h; (1f) dwelling for 66 h. The last temperature was held before the start of the next heating program

(sum time 24 days): (2a) heating to 450 °C in 2 h; (2b) dwelling for 48 h; (2c) heating to 550 °C in 12 h; (2d) dwelling for 504 h; (2e) cooling to 25 °C in 12 h. After the reaction, the whole ampoule was covered with a metal mirror, with the top 2 cm being more transparent. Most of the product was at the bottom of the ampoule. The ampoule was opened in the glovebox; the product mixture consisted of air-sensitive black crystals with high mechanical hardness. Yield: 15.91-16.27 g (98-99+ %, based on the sum mass of starting materials).

*Synthesis of [Ti(py)$_4$I$_2$]·2Py (**1**).* The complexation of TiI_2 and its separation were achieved by a Soxhlet extraction. *Assembly*: All glassware and the glass fibre extraction thimble were dried in the drying cabinet prior to use. The whole assembly (from bottom to the top, see SI Figure S16 for a photo): (a) heating mantle; (b) 500 mL flask; (c) cutoff adapter; (d) adapter with a hose connection in the middle; (e) 100 mL Soxhlet extractor; (f) reflux condenser; (g) adapter with a hose connection on the top, connecting the apparatus to an overpressure valve. The silicon grease was used only at the joint between the flask and the cutoff adapter. For every other connection, an NS Teflon sleeve was used. *Preparation*: Inside the glovebox, the ampoule from the synthesis of TiI_2 (in a mixture with an excess of Ti metal) was unsealed, and its whole contents were placed in the extraction thimble, which was then inserted inside the Soxhlet apparatus, which was then closed from both sides with a stopper and a cap. The bottom (below the Soxhlet extractor) and top (above the Soxhlet extractor) parts of the apparatus were closed with a stopper or cap and were thrice vacuumed and flushed with argon. Using the transfer needle, 250 mL of dry pyridine were added to the flask. The solvent was degassed using the "freeze-pump-thaw" technique three times. The cutoff adapter valve was closed. The remaining bottom section was vacuumed and flushed with argon once. Under the counterflow of argon (provided via connector (d)), the Soxhlet extractor was installed. Then, in a similar fashion, the condenser was installed. The whole apparatus (with the cutoff adapter (c) valve still closed) was vacuumed and flushed with argon twice. The connector (g) was opened to the overpressure valve, which was checked for functionality. The valve of the connector (d) was closed, and the cutoff valve (c) was opened. *Extraction*: the condenser was supplied with cooling water, and the heating mantle was turned to the maximum. The extraction was run overnight (for 21 h), with one extraction cycle taking about 12 minutes. The pyridine solution containing [Ti(py)$_4$I$_2$] is intensely blue coloured, appearing black at high concentration. The end of the extraction was recognised by the absence of colour in pyridine coming from the Soxhlet extractor. At last, the apparatus was supplied with an argon flow via connector (d) and the heat was turned off. The slight overpressure of the inert atmosphere on the apparatus was necessary, otherwise upon cooling, the air oxygen might come in, leading to its unwanted reaction with [Ti(py)$_4$I$_2$], which can be recognised by appearing green tint on the surface of the complex solution and brown to green tint of the powder. Once the reaction apparatus cooled down, the cutoff adapter (c) valve was closed. *Solvent removal by cold distillation:* The apparatus was disassembled, leaving the cutoff adapter on the flask. A hose adapter was placed on the top, connected to a cold trap and then to the vacuum gas manifold. The flask was placed in a room-temperature water bath. The connection to the vacuum gas manifold was vacuumed and filled with argon three times. Then, the cutoff adapter (c) was opened, and cold distillation was performed (≈4 h), with the cold trap cooled with liquid nitrogen. Finally, the flask with the cutoff valve was connected directly to the vacuum gas manifold, and the solid product was dried for 4 h at 25 °C and 0.001 mbar. After that, the flask under vacuum, with the cutoff valve closed, was transferred to the glovebox. The product was a black crystalline powder that had a deep blue colour once mortared. Yield: 37.47 g (97 %, based on the maximum yield of 50 mmol). Elemental analysis calculated (%) for $C_{30}H_{30}I_2N_6Ti$: C 46.42, H 3.90, N 10.83; found: C 46.34, H 3.89, N 10.96.

*Synthesis of [Ti(py)$_4$I$_2$] (**2**).* The synthesis of **2** was carried out in a similar fashion to that of **1**, with the steps of *Assembly*, *Preparation,* and *Extraction* being the same. *Solvent removal by cold distillation:* The apparatus was disassembled, leaving the cutoff adapter (valve was closed before the disassembly) on the flask. A hose adapter was placed on the top, connected to a cold trap and then to the vacuum gas manifold. The flask was placed in an oil bath at 30 °C. The connection to the vacuum gas manifold was vacuumed and filled with vacuum three times. Then, the cutoff adapter (c) was opened, and cold distillation was performed (≈6 h), with the cold trap cooled with liquid nitrogen. *Intercalated pyridine removal*: once the liquid pyridine was removed and only the solid product remained, the oil bath temperature was raised stepwise by 10 °C every 20 minutes to 80 °C. At this temperature, the product was dried under active vacuum for 3 h, with the cold trap emptied in between. Finally, the flask was cooled and transferred to the glovebox under vacuum with the cutoff valve closed. The product was a very dark blue powder. Yield: 30.45 g (99 %, based on the maximum yield of 50 mmol). Elemental analysis calculated (%) for $C_{20}H_{20}N_4I_2N_4Ti$: C 38.87, H 3.26, N 9.06; found: C 38.50, H 3.21, N 9.15.

*Synthesis of [Ti(bpy)$_2$I$_2$] (**3**).* Inside the glovebox, a stir bar, bpy (328 mg, 2.10 mmol), and [Ti(py)$_4$I$_2$] (**2**, 618 mg, 1.0 mmol) were placed in a Duran culture tube (outer diameter 12 mm, height 100 mm). To it, dry toluene (4 mL) was added upon stirring. Then, the reaction was stirred in an oil bath (outside the glovebox) at 70 °C for 17 h. Subsequently, the culture tube was centrifuged (4000 rpm, 12 min), and the solvent was removed with a syringe inside the glovebox. The powder product was washed twice with dry toluene (2 x 4 mL) inside the culture tube using the centrifuge to speed up the decantation; the solvent exchange was done in the glovebox. Prior to the last centrifugation, the stir bar was removed from the tube inside the glovebox. After that, the solvent was removed (also inside the glovebox), and the tube containing the product was placed in a special quick-fit, then dried under vacuum (using the vacuum gas manifold) at 50 °C (oil bath) for 30 minutes. The product was a black powder. Yield: 600 mg (98 %). Elemental analysis calculated (%) for $C_{20}H_{16}N_4I_2N_4Ti$: C 39.12, H 2.63, N 9.12; found: C 39.30, H 2.72, N 9.17.

Synthesis of a mixture of TiI$_2$ and TiI$_3$. This ampoule with the reaction mixture was prepared in a manner similar to the synthesis of TiI_2 (in a mixture with an excess of Ti metal), except for the ratio of starting compounds: 12.69 g of iodine (50.0 mmol) and 2.396 g titanium powder (3.595, 75.0 mmol) were used. Following temperature program was performed (sum time 32 days): (1a) heating to 130 °C in 2 h; (1b) dwelling for 46 h; (1c) heating to 180 °C in 2 h; (1d) dwelling for 46 h; (1e) heating to 350 °C in 6 h; (1f) dwelling for 190 h; (1g) heating to 400 °C in 2 h; (1h) dwelling for 474 h; followed by a natural cooling to room temperature. After this, the ampoule was removed from the oven and inspected: most of the solid was at the bottom, with only a few black needle crystals at the top. Several colours were observed in the solid reaction mixture, including a red tint, indicating incomplete reaction. The ampoule was placed back in the oven and the following temperature program was used (sum time 11.3 days): (2a) heating to 400 °C in 6 h; (2b) dwelling for 12 h; (2c) heating to 470 °C in 2 h; (2d) dwelling for 240 h; (2e) cooling to 25 °C in 12 h. On top of the ampoule, a net of long black needles formed; the solid on the bottom also consisted of black needles and came off the glass easily. The compound from the top of the ampoule was forced down to the bottom (by shaking the ampoule). Then, the ampoule was carefully heated with a heat gun (≈ 230 °C) with no fumes being observed (indicating absence of TiI_4 and I_2). The ampoule was opened in the glovebox; the product mixture consisted of air-sensitive black crystals with high mechanical hardness. Yield: 15.08 g (99+ %, based on the sum mass of starting materials).

S1.4. Single crystal synthesis

*Synthesis of [Ti(py)$_4$I$_2$]·2Py (**1**) single crystals.* Inside the glovebox, dry pyridine (2 mL) was added to 100 mg of the reaction mixture from the synthesis of TiI_2 (containing TiI_2 in a mixture with an excess of Ti metal), and the vial with this mixture was shaken several times within 1 h. After that, 1 mL of the suspension was filtered through a PTFE syringe filter. A portion of this filtrate (0.3 mL) was added to another 20 mg of TiI_2/Ti mixture in a Duran glass tube, which was then sealed under vacuum to make an ampoule. This ampoule was heated in a tube furnace according to the following heating program: heating to 150 °C in 1 h; dwelling for 6 h; cooling to 25 °C at 20 °C/h. Afterwards, the ampoule was unsealed inside the glovebox, and the solvent was removed. The dark crystals formed were prepared for measurement inside the glovebox and transferred using the Schlenk apparatus to the diffractometer for analysis.

*Synthesis of [Ti(py)$_4$I$_2$] (**2**) single crystals.* Inside the glovebox, a concentrated solution of [Ti(py)$_4$I$_2$]·2Py (**1**) in benzene was prepared by adding 2 mL of dry benzene to **1**, and then by filtering the suspension formed through a PTFE syringe filter. This filtrate (0.3 mL) was added to another 5 mg of **1** in a Duran glass tube, which was sealed under vacuum to make an ampoule without freezing the solvent. The ampoule was placed in a glass oven (Büchi) that was heated to 70 °C for 5 days. Afterwards, the ampoule was unsealed inside the glovebox, and the solvent was removed. The dark crystals formed were prepared for measurement inside the glovebox and transferred using the Schlenk apparatus to the diffractometer for analysis.

*Synthesis of [Ti(bpy)$_2$I$_2$] (**3**) single crystals.* Inside the glovebox, a stir bar, bpy (312 mg, 2.0 mmol), and the mixture of TiI_2/TiI_3 (302 mg) were placed in a Duran culture tube (outer diameter 12 mm, height 100 mm). To it, dry acetonitrile (3 mL) was added. Then, the reaction was stirred in an oil bath (outside the glovebox) at 80 °C for 43 h. Subsequently, the culture tube was centrifuged (4000 rpm, 20 min), and the solvent was removed with a syringe inside the glovebox. The tube containing the product was placed in a special quick-fit, dried under vacuum (using the vacuum gas manifold) for 30 minutes, and then returned to the glovebox. A portion of the resulting black powder (10 mg) was sealed under vacuum in an ampoule with a dry diethyl ether (0.5 mL) and a piece of lithium metal, which was slightly cut with a spatula for exposure. This ampoule was heated in a tube furnace according to the following heating program: heating to 100 °C in 1 h; dwelling for 12 h; cooling to 25 °C in 2 h. Afterwards, the ampoule was unsealed inside the glovebox, and the solvent and remaining lithium were removed. The dark, non-transparent crystals formed were prepared for measurement inside the glovebox and transferred using the Schlenk apparatus to the diffractometer for analysis.

*Synthesis of [Ti(bpy)$_2$I$_2$]I (**4**) single crystals.* The same black powder sample used for the synthesis of single crystals of **3** (obtained in the reaction between bpy and the mixture of TiI_2/TiI_3 in acetonitrile) was used here. A portion of this black powder (20 mg) was sealed under vacuum in an ampoule with dry acetonitrile (0.2 mL). The ampoule was placed in a glass oven (Büchi) that was heated to 80 °C for 5 days. Afterwards, the ampoule was unsealed inside the glovebox, and the solvent was removed. The orange crystals formed were taken outside the glovebox and analysed.

*Synthesis of [Ti(bpy)$_2$I$_2$]I·0.5bpy (**5**) single crystals.* Inside the glovebox, bpy (234 mg, 1.5 mmol) and the mixture of TiI_2/TiI_3 (60 mg) were placed in a Duran tube, which was then sealed under vacuum to make an ampoule. The ampoule was placed in a glass oven (Büchi) that was heated to 85 °C for 9 days. After that, the oven was rotated to a horizontal position, and the ampoule was placed so that its top was slightly sticking out of the oven; in this manner, the excess of bpy was removed by sublimation within several days. Afterwards, the ampoule was unsealed inside the glovebox. The dark block crystals formed were prepared for measurement inside the glovebox and then transferred under argon to the diffractometer for analysis.

*Synthesis of [Ti(bpy)$_2$ClI]I·CHCl$_3$ (**6**) single crystals.* Inside the glovebox, bpy (63 mg, 0.4 mmol) and a mixture of TiI_2/TiI_3 (60 mg) were placed together with dry chloroform (0.5 mL) in a Duran tube, which was then sealed under vacuum to make an ampoule. The ampoule was placed in a glass oven (Büchi) that was heated to 50 °C for 9 days. Afterwards, the ampoule was unsealed inside the glovebox, and the solvent was removed. The dark crystals formed were prepared for measurement inside the glovebox and then transferred under argon to the diffractometer for analysis.

Synthesis of TiI$_2$ single crystals. The crystals of TiI_2 were obtained in the reaction between Ti sponge (3-13 mm pieces, 1.436 g, 30.0 mmol) and iodine (7.614 g, 30.00 mmol) that was prepared similarly to the main synthesis of TiI_2 (in a mixture with an excess of Ti metal) reported here. To describe shortly, the reaction was heated in a tube furnace at 500 °C for 48 h, then at 600 °C for 96 h, then at 750 °C for 24 h, then at 700 °C for 96 h, with the ampoule taken out for inspection between each heating program. Afterwards, the ampoule was unsealed inside the glovebox. Black air-sensitive crystals with high mechanical hardness were prepared for the measurement inside the glovebox and transferred using the Schlenk apparatus to the diffractometer for analysis.

Synthesis of TiI$_3$ single crystals. Inside the glovebox, the mixture of TiI_2/TiI_3 (30 mg) and ZnI_2 (33 mg, 0.1 mmol) was placed in a self-made quartz tube (outer diameter 10 mm), which was then sealed under vacuum to make an ampoule. The reaction was heated in a tube furnace at 470 °C for 30 h. Afterwards, the ampoule was unsealed inside the glovebox. The molten cake of ZnI_2 also contained black needle crystals, which were prepared for the measurement inside the glovebox and transferred using the Schlenk apparatus to the diffractometer for analysis.

Synthesis of TiI$_4$ single crystals. Inside the glovebox, the mixture of TiI_2/TiI_3 (2.02 g) was filled in a self-made quartz tube (outer diameter 18 mm), which was fixed in a quick-fit to connect it to the vacuum gas manifold. The mixture was heated in a mantle first at 550 °C for 1 h, then at 650 °C for 2 h. Black block crystals formed inside the tube 2-3 cm above the edge of the heating mantle. They were transferred to the glovebox, weighed (0.84 g), and transferred under argon to the diffractometer for the analysis.

S2. Crystallographic data

S2.1. Comparison of measured and simulated powder X-ray diffraction patterns

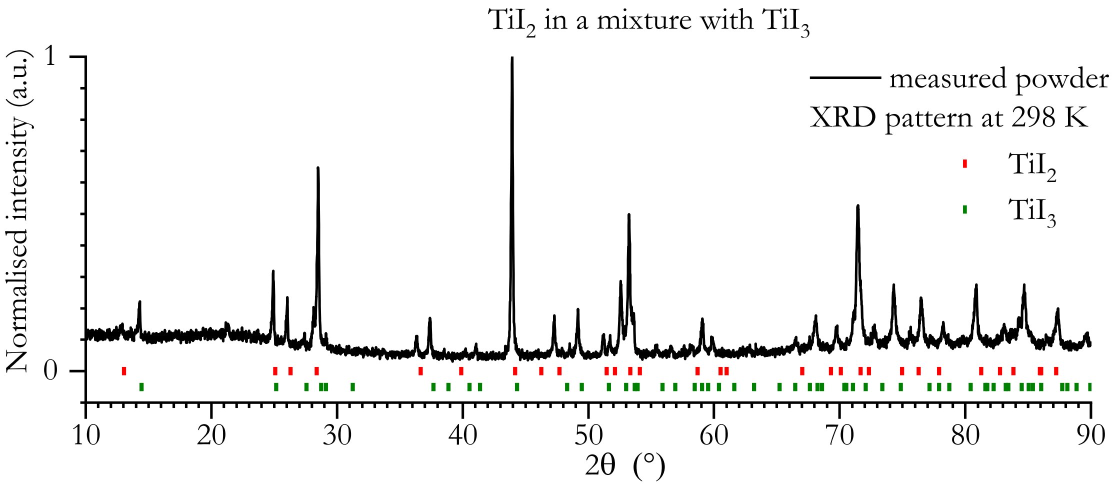

Figure S1. Experimental powder X-ray diffraction pattern of the reaction mixture from the synthesis of TiI_2 (in a mixture with TiI_3, molar ratio of Ti:I_2 used in the reaction 1:1). Compared to Bragg markers of TiI_2 (at 100 K, red top markers) and TiI_3 (at 100 K, green bottom markers).

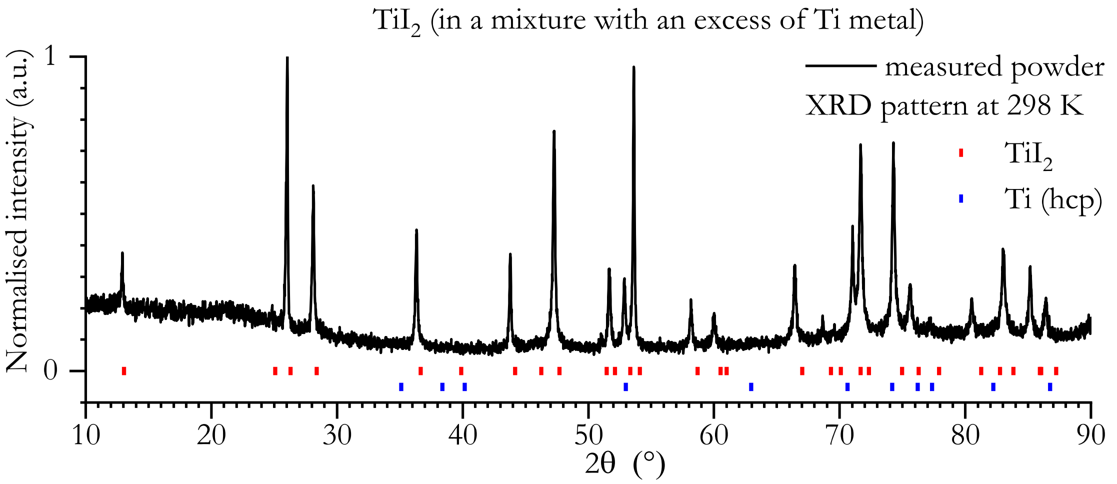

Figure S2. Experimental powder X-ray diffraction pattern of the reaction mixture from the synthesis of TiI_2 (in a mixture with an excess of Ti metal, molar ratio of Ti:I_2 used in the reaction 1.5:1). Compared to Bragg markers of TiI_2 (at 100 K, red top markers) and Ti (hcp, blue bottom markers).[73]

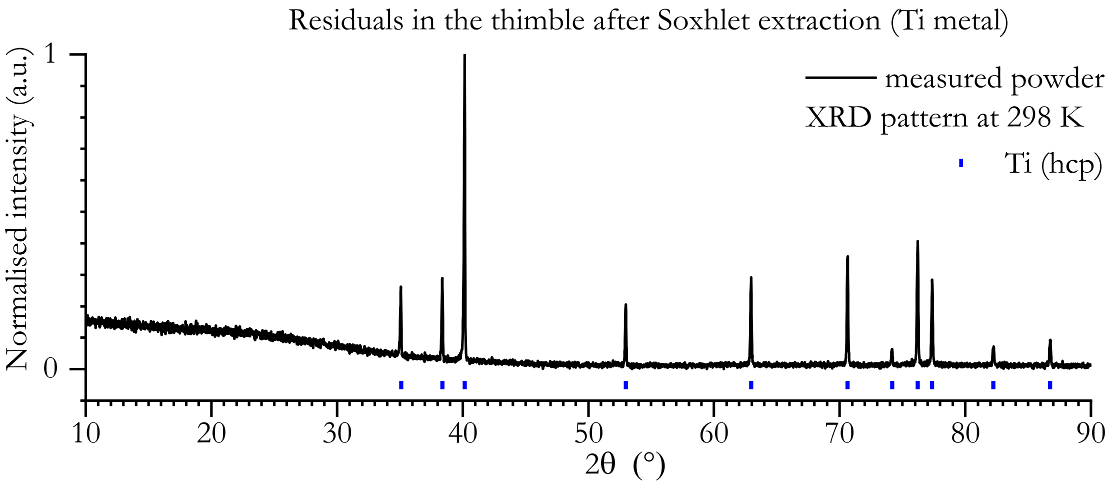

Figure S3. Experimental powder X-ray diffraction pattern of residuals in the thimble after Soxhlet extraction (from the synthesis of TiI_2 obtained in a mixture with an excess of Ti metal, molar ratio of Ti:I_2 used in the reaction 1.5:1). Compared to Bragg markers of Ti (hcp, blue bottom markers).[73]

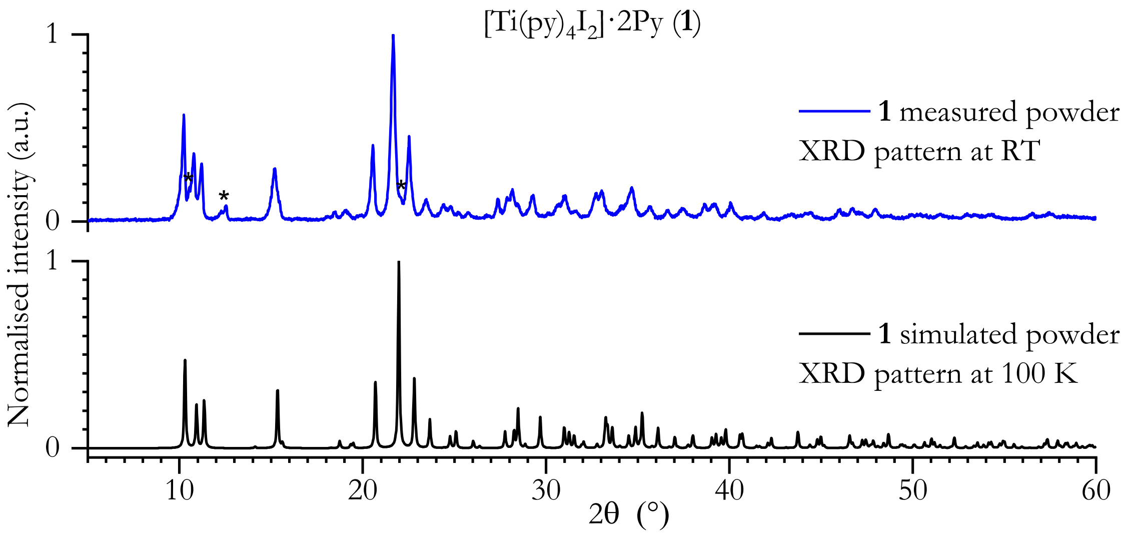

Figure S4. Comparison of experimental (top) and simulated (bottom) powder X-ray diffraction patterns of $[Ti(py)_4I_2]\cdot 2Py$ (**1**). Peaks and shoulders marked with a star (at 2θ angles 10.53, 12.51, 21.78) belong to **2** that forms during the sample preparation.

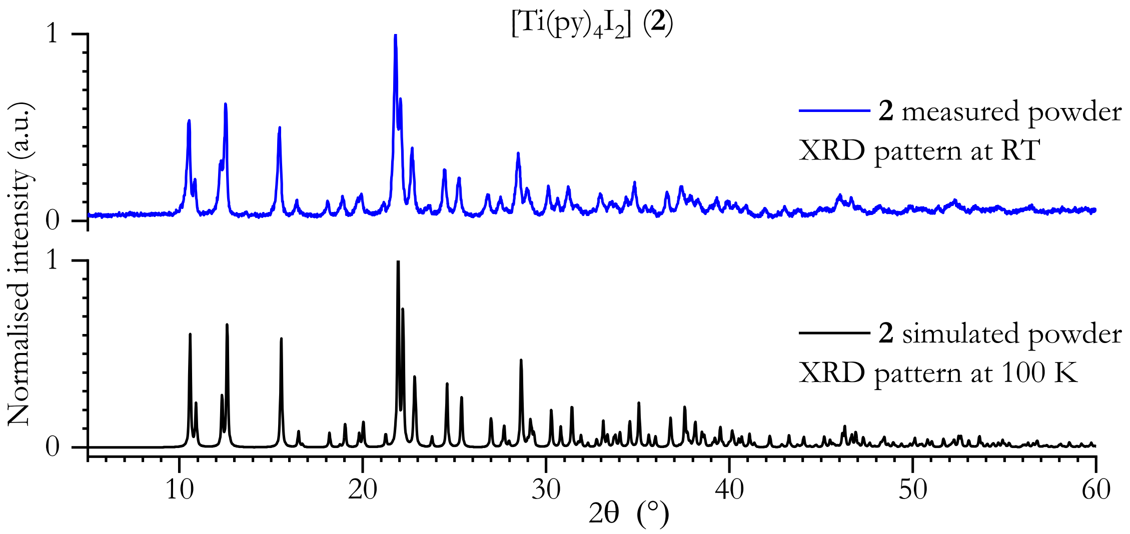

Figure S5. Comparison of experimental (top) and simulated (bottom) powder X-ray diffraction patterns of $[Ti(py)_4I_2]$ (**2**).

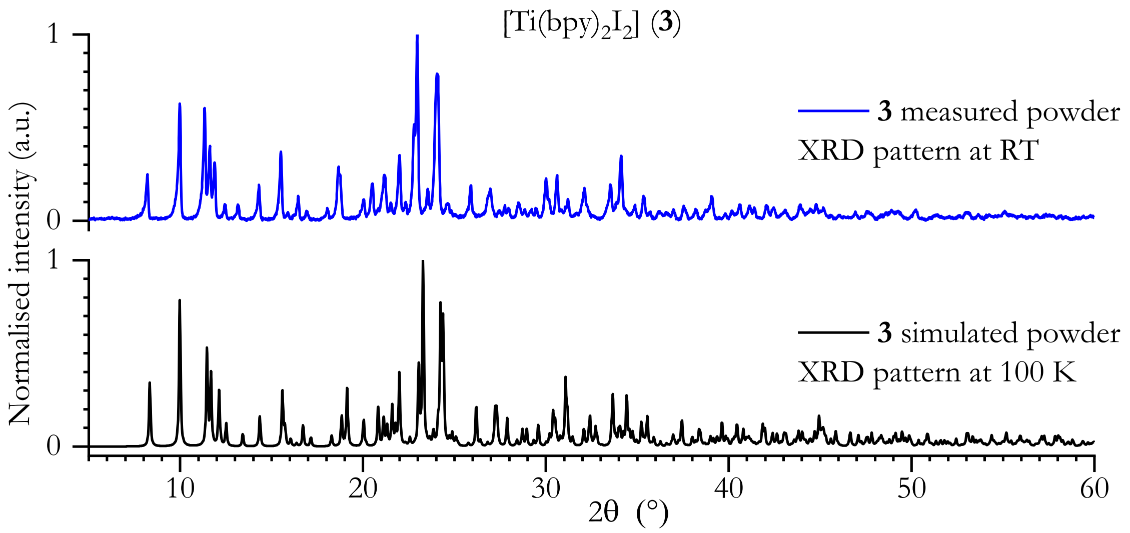

Figure S6. Comparison of experimental (top) and simulated (bottom) powder X-ray diffraction patterns of $[Ti(bpy)_2I_2]$ (**3**).

S2.2. Tables with X-ray crystallographic data

Table S1. Crystallographic data for $[Ti(py)_4I_2]\cdot 2Py$ (**1**), $[Ti(py)_4I_2]$ (**2**), and $[Ti(bpy)_2I_2]$ (**3**).

Compound	$[Ti(py)_4I_2]\cdot 2Py$ (**1**)	$[Ti(py)_4I_2]$ (**2**)	$[Ti(bpy)_2I_2]$ (**3**)
CCDC number	2513455	2513456	2513457
Empirical formula	$C_{30}H_{30}I_2N_6Ti$	$C_{20}H_{20}I_2N_4Ti$	$C_{20}H_{16}I_2N_4Ti$
M_r / g·mol^{-1}	776.30	618.10	614.07
Crystal system	orthorhombic	orthorhombic	monoclinic
Space group	*Ccca*	*Pbcn*	$P2_1/c$
a / Å	11.3419(3)	9.7496(3)	8.9123(6)
b / Å	15.5823(5)	16.2160(5)	14.5812(11)
c / Å	17.1566(6)	14.0179(5)	15.5196(12)
α / °	90	90	90
β / °	90	90	96.388(3)
γ / °	90	90	90
V / Å^3	3032.14(16)	2216.22(12)	2004.3(3)
Z	4	4	4
ρ_{calc} / g·cm^{-3}	1.701	1.852	2.035
F(000)	1520	1184	1168
μ / mm^{-1}	2.348	25.233	3.520
T / K	100	200	100
θ_{min}	2.374	5.294	2.299
θ_{max}	31.025	70.023	27.484
X-ray radiation	Mo-K$_\alpha$, λ = 0.71073 Å	Cu-K$_\alpha$, λ = 1.54178 Å	Mo-K$_\alpha$, λ = 0.71073 Å
Collected reflections	42958	33537	52923
Unique reflections	2429	2106	4598
No. of parameters	93	125	244
R_1 [a] / wR_2 [b] ($I > 2\sigma(I)$)	0.0201 / 0.0537	0.0387 / 0.1080	0.0340 / 0.0753
R_1 [a] / wR_2 [b] (all data)	0.0290 / 0.0575	0.0491 / 0.1172	0.0565 / 0.0852
GOF on F^2	1.058	1.104	1.072
Largest residual electron density peaks / e·Å^{-3}	0.457 / -0.879	1.384 / -0.784	0.680 / -1.307

[a] $R_1 = \frac{\Sigma||F_o|-|F_c||}{\Sigma|F_o|}$. [b] $wR_2 = \sqrt{\frac{\Sigma w(F_o^2-F_c^2)^2}{\Sigma w(F_o^2)^2}}$.

Table S2. Crystallographic data for $[Ti(bpy)_2I_2]I$ (**4**), $[Ti(bpy)_2I_2]I{\cdot}bpy$ (**5**), and $[Ti(bpy)_2ClI]I{\cdot}CHCl_3$ (**6**).

Compound	$[Ti(bpy)_2I_2]I$ (**4**)	$[Ti(bpy)_2I_2]I{\cdot}0.5bpy$ (**5**)	$[Ti(bpy)_2ClI]I{\cdot}CHCl_3$ (**6**)
CCDC number	2513458	2513459	2513460
Empirical formula	$C_{20}H_{16}I_3N_4Ti$	$C_{25}H_{20}I_3N_5$ Ti	$C_{21}H_{17}Cl_4I_2N_4Ti$
M_r / g·mol^{-1}	740.97	819.06	768.89
Crystal system	triclinic	monoclinic	triclinic
Space group	$P\overline{1}$	$I2/a$	$P\overline{1}$
a / Å	8.3536(10)	20.0083(13)	8.5868(7)
b / Å	10.4869(13)	12.7076(8)	12.7295(11)
c / Å	14.1070(16)	23.146(2)	14.0386(11)
α / °	96.715(4)	90	112.585(3)
β / °	99.371(4)	112.128(2)	90.346(3)
γ / °	107.954(4)	90	107.523(3)
V / Å^3	1141.4(2)	5451.5(7)	1338.11(19)
Z	2	8	2
ρ_{calc} / g·cm^{-3}	2.156	1.996	1.908
F(000)	690	3088	734
μ / mm^{-1}	4.445	3.734	3.043
T / K	120	150	150
θ_{min}	2.075	1.943	2.511
θ_{max}	27.487	26.732	29.586
X-ray radiation	Mo-K_α, λ = 0.71073 Å	Mo-K_α, λ = 0.71073 Å	Mo-K_α, λ = 0.71073 Å
Collected reflections	57761	74413	70422
Unique reflections	5236	5793	7487
No. of parameters	253	327	308
R_1 [a] / wR_2 [b] ($I > 2\sigma(I)$)	0.0454 / 0.0910	0.0425 / 0.1120	0.0354 / 0.0787
R_1 [a] / wR_2 [b] (all data)	0.0817 / 0.1073	0.0558 / 0.1216	0.0475 / 0.0847
GOF on F^2	1.030	1.040	1.084
Largest residual electron density peaks / e·Å^{-3}	1.075 / -1.310	1.829 / -1.375	1.295 / -1.290

[a] $R_1 = \frac{\Sigma||F_o|-|F_c||}{\Sigma|F_o|}$. [b] $wR_2 = \sqrt{\frac{\Sigma w(F_o^2-F_c^2)^2}{\Sigma w(F_o^2)^2}}$.

Table S3. Crystallographic data for TiI_2, TiI_3, TiI_4.

Compound	TiI_2	TiI_3	TiI_4
CCDC number	2513461	2513462	2513463
Empirical formula	TiI_2	TiI_3	TiI_4
M_r / g·mol^{-1}	301.70	428.60	555.50
Crystal system	trigonal	hexagonal	cubic
Space group	$P\bar{3}m1$	$P6_3/mcm$	$Pa\bar{3}$
a / Å	4.0975(2)	7.0739(2)	11.8965(10)
b / Å	4.0975(2)	7.0739(2)	11.8965(10)
c / Å	6.7744(7)	6.4655(3)	11.8965(10)
α / °	90	90	90
β / °	90	90	90
γ / °	120	120	90
V / Å^3	98.501(14)	280.19(2)	1683.7(4)
Z	1	2	8
ρ_{calc} / g·cm^{-3}	5.086	5.080	4.383
F(000)	128	362	1872
μ / mm^{-1}	17.584	17.881	15.575
T / K	100	100	100
θ_{min}	3.007	3.325	2.966
θ_{max}	28.172	41.015	42.011
X-ray radiation	Mo-K$_\alpha$, λ = 0.71073 pm	Mo-K$_\alpha$, λ = 0.71073 pm	Mo-K$_\alpha$, λ = 0.71073 pm
Collected reflections	1902	7920	49552
Unique reflections	119	363	1978
No. of parameters	7	7	17
R_1 [a] / wR_2 [b] ($I > 2\sigma(I)$)	0.0202 / 0.0455	0.0191 / 0.0435	0.0328 / 0.0698
R_1 [a] / wR_2 [b] (all data)	0.0202 / 0.0455	0.0224 / 0.0446	0.0853 / 0.0838
GOF on F^2	1.312	1.117	1.008
Largest residual electron density peaks / e·Å^{-3}	1.298 / -1.668	1.193 / -1.083	1.390 / -1.260

[a] $R_1 = \frac{\Sigma||F_o|-|F_c||}{\Sigma|F_o|}$. [b] $wR_2 = \sqrt{\frac{\Sigma w(F_o^2-F_c^2)^2}{\Sigma w(F_o^2)^2}}$.

S2.3. Tables with selected interatomic distances and angles

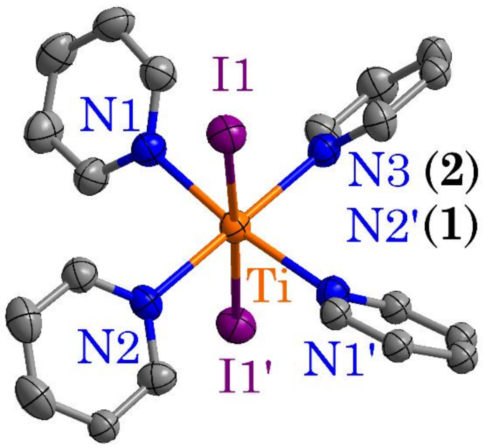

Figure S7. Selected view of X-ray crystal structure of [Ti(py)$_4$I$_2$] (**2**). Thermal ellipsoids describe a 50 % probability level of the atoms (Ti orange, I violet, C grey, N blue); hydrogen atoms are omitted.

Table S4. Selected interatomic distances (Å) and angles (°) of [Ti(py)$_4$I$_2$]·2Py (**1**) and [Ti(py)$_4$I$_2$] (**2**).

	[Ti(py)$_4$I$_2$]·2Py (**1**)		[Ti(py)$_4$I$_2$] (**2**)
Ti-N1	2.2433(18)	Ti-N1	2.260(4)
Ti-N2	2.2782(18)	Ti-N2	2.248(5)
Ti-N2'	2.2782(18)	Ti-N3	2.248(6)
Ti-N1'	2.2433(18)	Ti-N1'	2.260(4)
Ti-I1	2.9259(2)	Ti-I1	2.8960(3)
Ti-I1'	2.9259(2)	Ti-I1'	2.8961(3)
N1-Ti-N2	90	N1-Ti-N2	93.73(11)
N1-Ti-N2'	90	N1-Ti-N3	86.27(11)
N1-Ti-N1'	180	N1-Ti-N1'	172.5(2)
N1-Ti-I1	90	N1-Ti-I1	89.34(11)
N1-Ti-I1'	90	N1-Ti-I1'	90.90(11)
N2-Ti-N2'	180	N2-Ti-N3	180
N2-Ti-N1'	90	N2-Ti-N1'	93.73(11)
N2-Ti-I1	90	N2-Ti-I1	88.14(2)
N2-Ti-I1'	90	N2-Ti-I1'	88.14(2)
N2'-Ti-N1'	90	N3-Ti-N1'	86.27(11)
N2'-Ti-I1	90	N3-Ti-I1	91.86(2)
N2'-Ti-I1'	90	N3-Ti-I1'	91.86(2)
N1'-Ti-I1	90	N1'-Ti-I1	90.90(11)
N1'-Ti-I1'	90	N1'-Ti-I1'	89.34(11)
I1-Ti-I1'	180	I1-Ti-I1'	176.28(4)

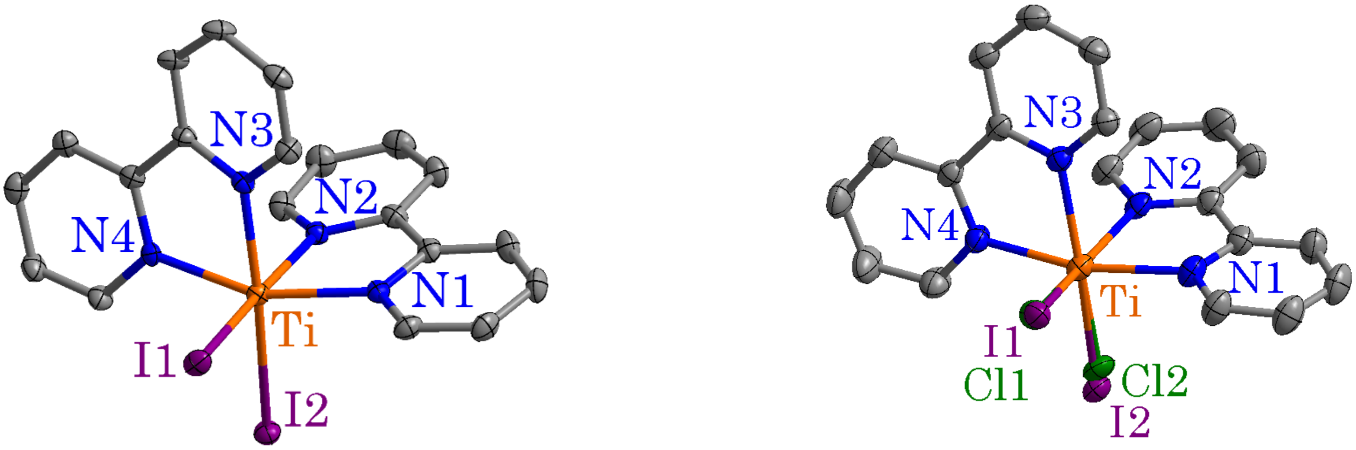

Figure S8. Selected views of X-ray crystal structures of $[Ti(bpy)_2I_2]$ (**3**, left), and $[Ti(bpy)_2ClI]I{\cdot}CHCl_3$ (**6**, right). Thermal ellipsoids describe a 50 % probability level of the atoms (Ti orange, I violet, Cl green, C grey, N blue); hydrogen atoms are omitted.

Table S5. Selected interatomic distances (Å) and angles (°) of $[Ti(bpy)_2I_2]$ (**3**), $[Ti(bpy)_2I_2]I$ (**4**), $[Ti(bpy)_2I_2]I{\cdot}bpy$ (**5**), and $[Ti(bpy)_2ClI]I{\cdot}CHCl_3$ (**6**).

	$[Ti(bpy)_2I_2]$ (**3**)	$[Ti(bpy)_2I_2]I$ (**4**)	$[Ti(bpy)_2I_2]I{\cdot}0.5bpy$ (**5**)		$[Ti(bpy)_2ClI]I{\cdot}CHCl_3$ (**6**)
Ti-N1	2.139(4)	2.160(6)	2.167(5)	Ti-N1	2.178(3)
Ti-N2	2.142(4)	2.179(6)	2.192(5)	Ti-N2	2.206(3)
Ti-N3	2.109(4)	2.190(6)	2.220(6)	Ti-N3	2.174(3)
Ti-N4	2.116(4)	2.172(6)	2.164(5)	Ti-N4	2.177(3)
Ti-I1	2.7572(9)	2.6995(14)	2.7003(11)	Ti-I1 / Ti-Cl1	2.649(3) / 2.352(6)
Ti-I2	2.7705(9)	2.6935(14)	2.7056(12)	Ti-I2 / Ti-Cl2	2.7719(8) / 2.332(5)
N1-Ti-N2	74.19(15)	74.2(2)	74.32(18)	N1-Ti-N2	74.1(1)
N1-Ti-N3	89.31(15)	88.6(2)	87.1(2)	N1-Ti-N3	96.69(10)
N1-Ti-N4	158.07(16)	157.9(2)	155.6(2)	N1-Ti-N4	165.15(11)
N1-Ti-I1	98.67(11)	98.74(17)	103.01(14)	N1-Ti-I1	92.40(12)
N1-Ti-I2	95.99(11)	96.65(16)	96.34(14)	N1-Ti-I2	90.88(8)
N2-Ti-N3	84.37(15)	81.5(2)	87.12(19)	N2-Ti-N3	79.03(10)
N2-Ti-N4	88.69(15)	89.0(2)	88.68(19)	N2-Ti-N4	92.14(10)
N2-Ti-I1	171.37(11)	167.90(17)	176.14(15)	N2-Ti-I1	160.67(11)
N2-Ti-I2	90.66(11)	92.93(16)	88.88(14)	N2-Ti-I2	89.40(7)
N3-Ti-N4	75.19(16)	74.5(2)	74.5(2)	N3-Ti-N4	74.77(10)
N3-Ti-I1	90.79(11)	88.58(16)	89.97(14)	N3-Ti-I1	89.12(11)
N3-Ti-I2	171.49(11)	171.01(17)	173.83(16)	N3-Ti-I2	163.82(7)
N4-Ti-I1	96.99(11)	95.03(17)	93.00(13)	N4-Ti-I1	99.48(12)
N4-Ti-I2	97.85(11)	98.49(17)	100.73(17)	N4-Ti-I2	94.61(8)
I1-Ti-I2	94.97(3)	97.74(4)	94.23(4)	I1-Ti-I2	104.89(9)

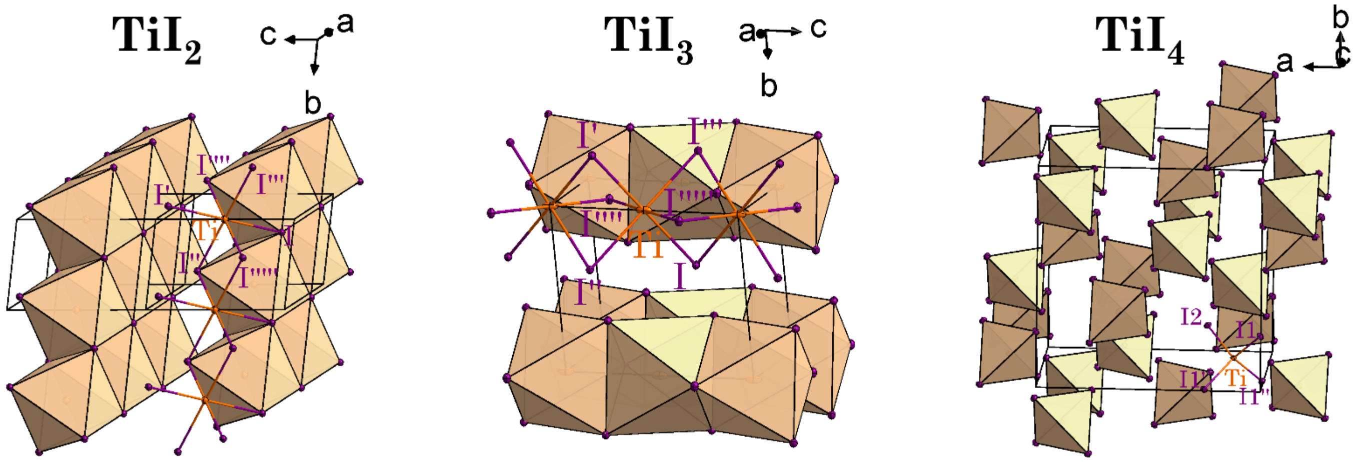

Figure S9. Selected views of X-ray crystal structures of TiI_2 (left), TiI_3 (middle), and TiI_4 (right). Thermal ellipsoids describe a 50 % probability level of the atoms (Ti orange, I violet). Coordination polyhedra of titanium are presented in light orange for clarity.

Table S6. Selected interatomic distances (Å) and angles (°) of TiI_2, TiI_3, TiI_4.

	TiI_2	TiI_3		TiI_4
Ti-I	2.8899(4)	2.7783(2)	Ti-I1	2.5327(6)
I-Ti-I'	180	180	Ti-I2	2.5363(3)
I''-Ti-I'''	180	180	I1-Ti-I1'	109.426(12)
I''''-Ti-I'''''	180	180	I1-Ti-I1''	109.426(12)
I-Ti-I''	89.705(14)	90.442(4)	I1-Ti-I2	109.517(12)
I-Ti-I'''	90.295(14)	89.558(4)	I1'-Ti-I1''	109.426(12)
I-Ti-I''''	89.705(14)	90.442(4)	I1'-Ti-I2	109.516(12)
I-Ti-I'''''	90.295(14)	89.558(4)	I1''-Ti-I2	109.516(12)
I'-Ti-I''	90.295(14)	89.558(4)		
I'-Ti-I'''	89.705(14)	90.442(4)		
I'-Ti-I''''	90.295(14)	89.558(4)		
I'-Ti-I'''''	89.705(14)	90.442(4)		
I''-Ti-I''''	90.295(14)	89.558(4)		
I''-Ti-I'''''	89.705(14)	90.442(4)		
I'''-Ti-I''''	89.705(14)	90.442(4)		
I'''-Ti-I'''''	90.295(14)	89.558(4)		

S3. UV-Vis-NIR absorbance spectra

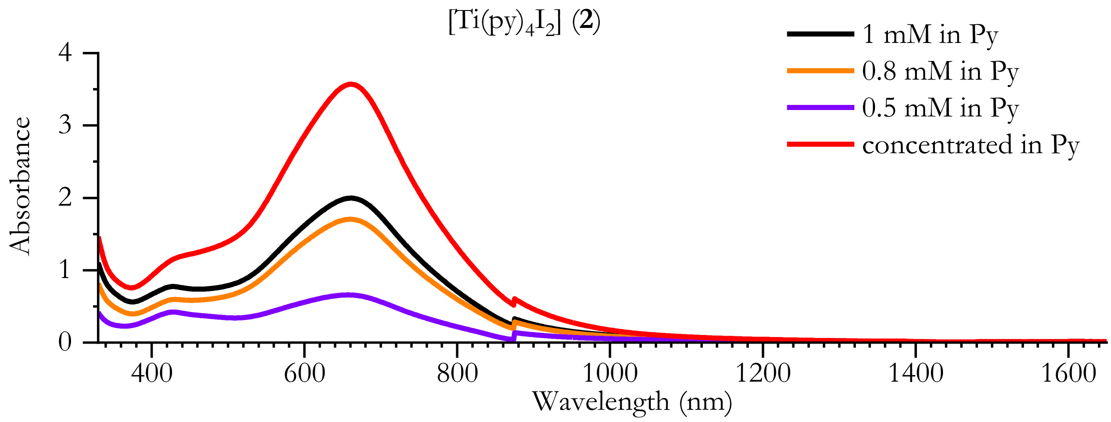

Figure S10. UV-Vis-NIR absorbance spectra of $[Ti(py)_4I_2]$ (**2**), recorded for 1 mM, 0.8 mM, 0.5 mM and concentrated solutions in dry pyridine in 10 mm cuvettes.

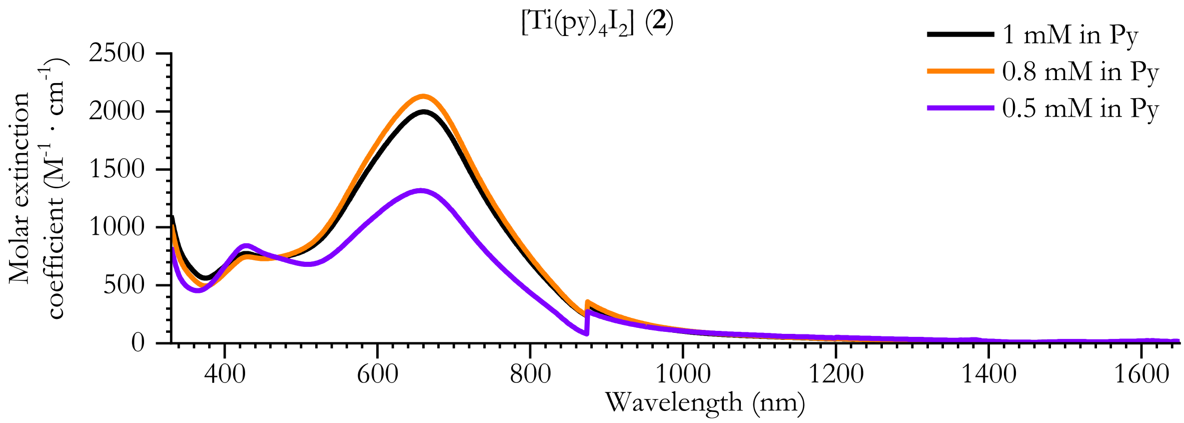

Figure S11. UV-Vis-NIR absorbance spectra of $[Ti(py)_4I_2]$ (**2**) of 1 mM, 0.8 mM, and 0.5 mM solutions, recalculated to molar extinction coefficient using Beer-Lambert Law.

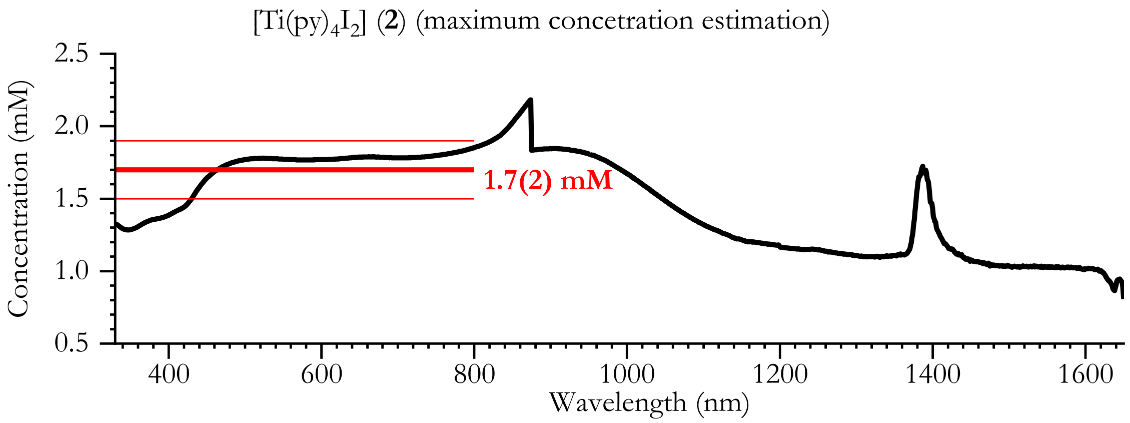

Figure S12. Estimation of the maximum concentration of **2** in pyridine. For this, the absorbance spectra of the concentrated solution (shown in Figure S10) was divided by the molar extinction coefficient of **2** (calculated for a 1 mM solution, shown in Figure S11) according to Beer-Lambert Law. The average value of concentration was calculated for the range 330-800 nm, corresponding to ≈1 mg · mL^{-1}.

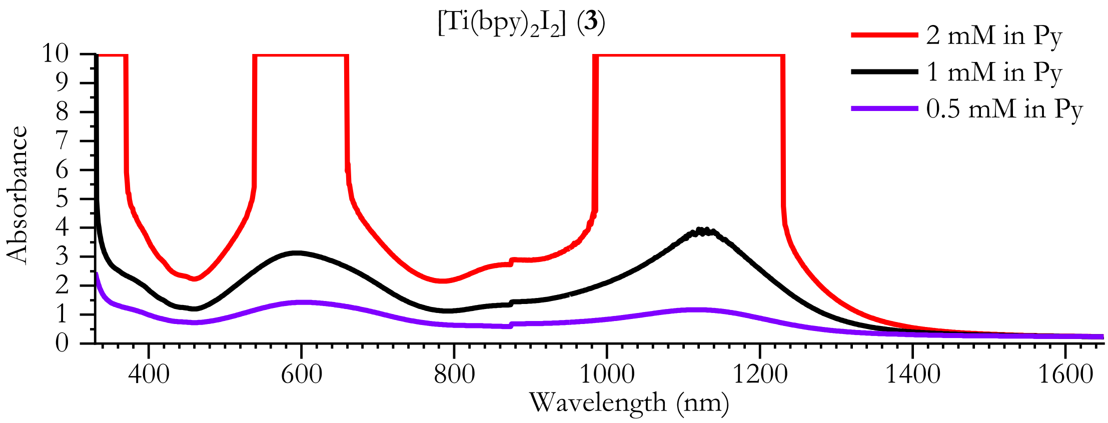

Figure S13. UV-Vis-NIR absorbance spectra of [Ti(bpy)$_2$I$_2$] (**3**), recorded for 2 mM, 1 mM, 0.5 mM and concentrated solutions in dry pyridine in 10 mm cuvettes.

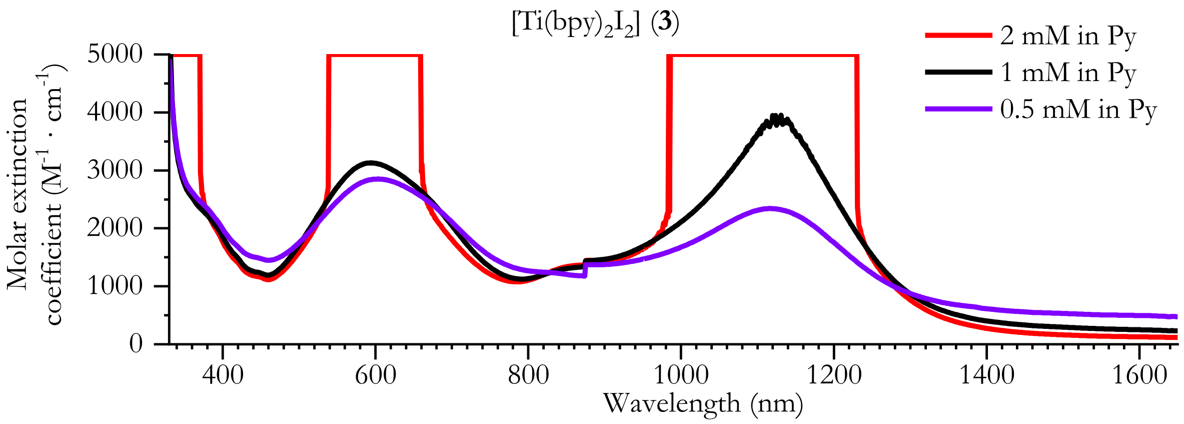

Figure S14. UV-Vis-NIR absorbance spectra of [Ti(bpy)$_2$I$_2$] (**3**) of 2 mM, 1 mM, and 0.5 mM solutions, recalculated to molar extinction coefficient using Beer-Lambert Law.

S4. Photos of selected experimental setup and reaction of [Ti(py)$_4$I$_2$] (2) solution with air

S4.1. Photo of the experimental setup used for the complexation/Soxhlet extraction of [Ti(py)$_4$I$_2$]

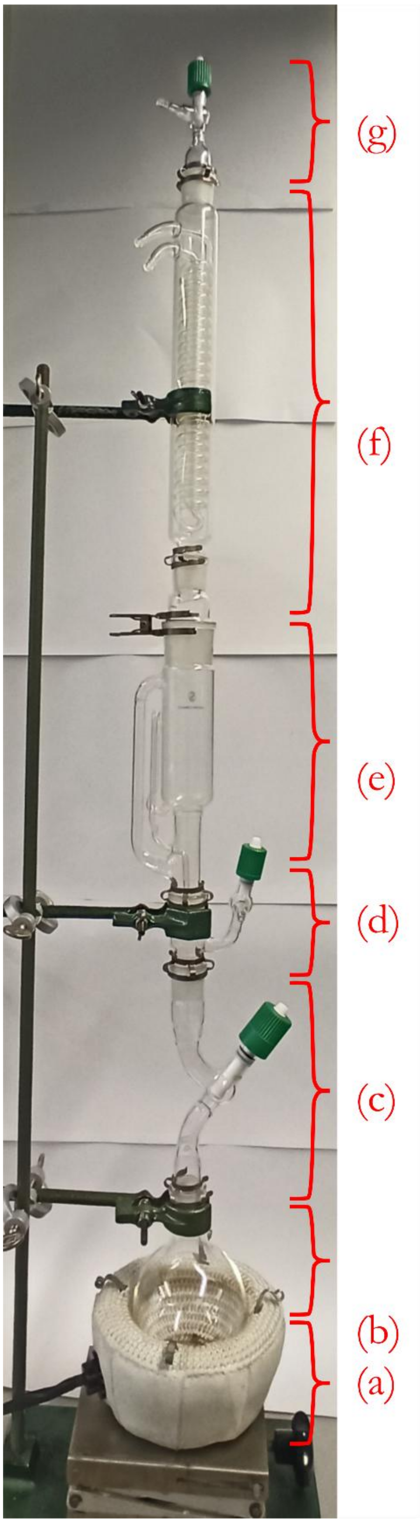

Figure S15. The assembly used in the synthesis of [Ti(py)$_4$I$_2$]·2Py (**1**) and [Ti(py)$_4$I$_2$] (**2**): (a) heating mantle; (b) 500 mL flask; (c) cutoff adapter; (d) adapter with a hose connection in the middle; (e) 100 mL Soxhlet extractor; (f) reflux condenser; (g) adapter with a hose connection on the top, connecting the apparatus to an overpressure valve.

S4.2. Reaction of $[Ti(py)_4I_2]$ (**2**) solution with air

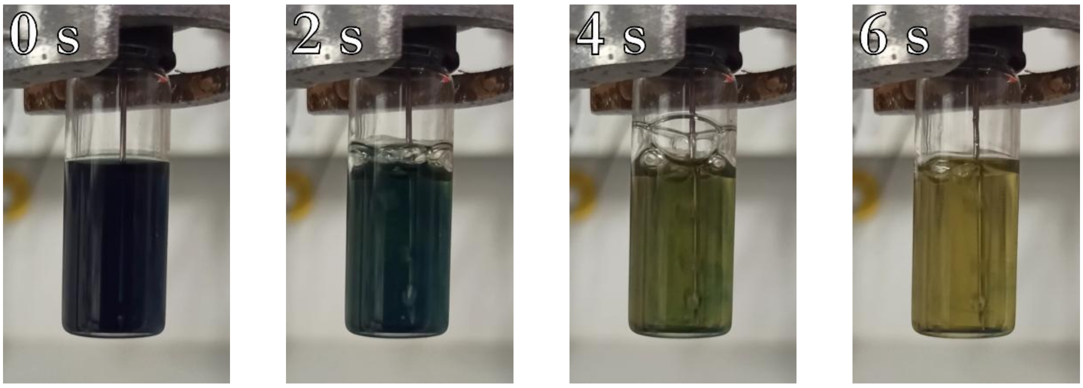

Figure S16. To a concentrated solution of $[Ti(py)_4I_2]$ (**2**) in pyridine (≈1 mg · mL^{-1}, 3 mL), air was added with a syringe, altogether ≈4 mL of air.
Left: solution before adding air; the solution is dark blue.
Middle left: solution two seconds after the addition of the air has started, a slight green tint is noticeable.
Middle right: solution four seconds after the addition of the air started, yellow/green coloured solution, a significant amount of **2** has already reacted.
Right: solution six seconds after the addition of the air started, yellow coloured solution, the reaction is practically complete.

S5. References

[15] J. D. Fast, "The preparation of pure titanium iodides" *Recl. Trav. Chim. Pays-Bas* **1939**, *58*, 174–180.

[16] W. Klemm, L. Grimm, "Zur Kenntnis der Dihalogenide des Titans und Vanadins" *Z. Anorg. Allg. Chem.* **1942**, *249*, 198–208.

[70] G. M. Sheldrick, "SHELXT – Integrated space-group and crystal-structure determination" *Acta Crystallogr., Sect. A: Found. Adv.* **2015**, *71*, 3–8.

[71] G. M. Sheldrick, "Crystal structure refinement with SHELXL" *Acta Crystallogr., Sect. C: Struct. Chem.* **2015**, 71, 3–8.

[72] C. B. Hübschle, G. M. Sheldrick, B. Dittrich, "ShelXle: A Qt graphical user interface for SHELXL" *J. Appl. Crystallogr.* **2011**, *44*, 1281–1284.

[73] R. W. G. Wyckoff, *Crystal Structures*, Interscience Publishers, **1963**.
